# Prevalence and Emergence of Extended-Spectrum Cephalosporin-, Carbapenem- and Colistin-Resistant Gram Negative Bacteria of Animal Origin in the Mediterranean Basin

**DOI:** 10.3389/fmicb.2018.02299

**Published:** 2018-09-28

**Authors:** Iman Dandachi, Selma Chabou, Ziad Daoud, Jean-Marc Rolain

**Affiliations:** ^1^IRD, APHM, MEPHI, IHU-Méditerranée Infection, Aix Marseille Université, Marseille, France; ^2^Clinical Microbiology Laboratory, Faculty of Medicine and Medical Sciences, University of Balamand, Beirut, Lebanon

**Keywords:** ESBL, carbapenemase, *mcr-1*, Mediterranean, livestock

## Abstract

In recent years, extended ESBL and carbapenemase producing Gram negative bacteria have become widespread in hospitals, community settings and the environment. This has been triggered by the few therapeutic options left when infections with these multi-drug resistant organisms occur. The emergence of resistance to colistin, the last therapeutic option against carbapenem-resistant bacteria, worsened the situation. Recently, animals were regarded as potent antimicrobial reservoir and a possible source of infection to humans. Enteric Gram negative bacteria in animals can be easily transmitted to humans by direct contact or indirectly through the handling and consumption of undercooked/uncooked animal products. In the Mediterranean basin, little is known about the current overall epidemiology of multi-drug resistant bacteria in livestock, companion, and domestic animals. This review describes the current epidemiology of ESBL, carbapenemase producers and colistin resistant bacteria of animal origin in this region of the world. The CTX-M group 1 seems to prevail in animals in this area, followed by SHV-12 and CTX-M group 9. The dissemination of carbapenemase producers and colistin resistance remains low. Isolated multi-drug resistant bacteria were often co-resistant to non-beta-lactam antibiotics, frequently used in veterinary medicine as treatment, growth promoters, prophylaxis and in human medicine for therapeutic purposes. Antibiotics used in veterinary medicine in this area include mainly tetracycline, aminoglycosides, fluoroquinolones, and polymyxins. Indeed, it appears that the emergence of ESBL and carbapenemase producers in animals is not related to the use of beta-lactam antibiotics but is, rather, due to the co-selective pressure applied by the over usage of non-beta-lactams. The level of antibiotic consumption in animals should be, therefore, re-considered in the Mediterranean area especially in North Africa and western Asia where no accurate data are available about the level of antibiotic consumption in animals.

## Background

Antimicrobial resistance is an emerging and rapidly evolving phenomenon. This phenomenon is currently observed in all bacterial species including clinically important Gram negative bacilli (GNB) (Rubin and Pitout, 2014). Gram negative bacilli, “enterobacteriaceae and non-fermenters” are normal inhabitants of the human intestinal microflora (Vaishnavi, 2013); they are responsible for the most common hospital and community acquired infections. Antibiotic resistance in GNB is mediated by target drug modification (Lambert, 2005), changes in bacterial cell permeability (Delcour, 2009) and, most importantly, the production of hydrolyzing enzymes, namely beta-lactamases. The most common beta-lactamases which are now widespread include the extended spectrum beta-lactamases (ESBL) (SHV, TEM, OXA, and CTX-M types), AmpC beta-lactamases, and carbapenemases (MBL, KPC, and class D oxacillinases) (Giedraitiene et al., 2011; Poirel et al., 2011). These enzymes provide the bacterium with resistance toward the majority of therapeutic options available in the clinical market. Furthermore, resistance determinants of these enzymes are often located on plasmids carrying resistance genes to other non-beta-lactam antibiotics, thus further limiting treatment options (Guerra et al., 2014).

The emergence of colistin resistance in GNB is another concern. Colistin belongs to the polymyxin group of polypeptide antibiotics (Olaitan et al., 2014a). Previously abandoned due to its nephrotoxicity and neurotoxicity, it is now in use once again and is considered to be the last resort antimicrobial agent against carbapenem resistant GNB (Kempf et al., 2013). Colistin resistance can be mediated either by the acquisition of the plasmid mediated “*mcr*” gene or by chromosomal mutations that lead to modification of the lipid A moiety of lipopolysaccharide (LPS), which is considered the primary target of colistin in Gram negative bacilli (Baron et al., 2016).

It is currently known that, in addition to the human intestinal microflora, resistant GNB can be found in water, soil, and fecal animal matter (Verraes et al., 2013). In fact, there is increasing evidence that animals constitute a potent reservoir of resistant GNB (Ewers et al., 2012). This is mainly due to the over- and misuse of antibiotics in veterinary medicine (Guerra et al., 2014): antibiotics are not only prescribed for treatment but are also administered for disease prevention and growth promotion (Economou and Gousia, 2015). Although studies have shown that the direct threat of resistant GNB to human health is still controversial (Olsen et al., 2014), the wide dissemination of these resistant organisms is worrying due to their ease of transmission (Rolain, 2013) and their high potential contribution to the spread of bacterial resistance across all ecosystems (Pomba et al., 2017). In this review, we attempt to describe the epidemiology of ESBL, AmpC and carbapenemase producing GNB of animal origin in the Mediterranean region. Colistin resistance in GNB in the same area is also described. The Mediterranean basin is a region of the world that compromises a wide diversity of populations. It includes five Asian countries (Cyprus, Israel, Lebanon, Syria, and Turkey), eleven European countries (Albania, Bosnia, Croatia, France, Greece, Herzegovina, Italy, Monaco, Montenegro, Slovenia, and Spain) and five African countries (Algeria, Egypt, Libya, Morocco, and Tunisia).

## Distribution of ESBLs and AmpC producers in animals

### Chicken and food of poultry origin

Poultry production is a complex system in the food and agricultural industry. It includes breeding chickens for meat and eggs. Chickens are kept either as a “breeding flock” or as a “broiler flock” for human consumption. Along with eggs, broilers are traded and transported across different countries around the world (Dierikx et al., 2013). This trade results in a vulnerable system that can be hacked by multi-drug resistant organisms (MDRO), i.e., once a MDRO is introduced into the production chain, it can be transferred internationally. This is why the dissemination of ESBL and AmpC-producing GNB, recently extensively reported in chicken farms (Blaak et al., 2015) is worrying, as these can contribute to not only local but global dissemination of antimicrobial resistance (Dierikx et al., 2013). Studies have shown that the carriage of ESBL and AmpC producers in chicken is persistent (Huijbers et al., 2016). ESBL and AmpC producers are isolated from grandparent breeding stock (Nilsson et al., 2014), broiler chickens (Reich et al., 2013), retail meat (Choi et al., 2015), and at the slaughterhouses (Maciuca et al., 2015).

In the Mediterranean basin, the first detection of ESBL in chicken dates back to 2000 in Greece, when a CTX-M-32 harboring *Salmonella enterica* was isolated from poultry end products (Politi et al., 2005). Since then, many studies have reported the emergence of ESBL in poultry in the Mediterranean area. In Italy for instance, the first ESBL reported was a case of SHV-12 detected in *Salmonella* spp (Chiaretto et al., 2008). *Salmonella infantis* species harboring CTX-M-1 were later isolated in 2011 from broiler chicken flocks. These strains led to human infection in Italy in 2013–2014 (Franco et al., 2015). In both studies, isolated strains were co-resistant to non-beta-lactam antibiotics, notably nalidixic acid, sulfonamide, trimethoprim, and tetracyclines. According to the European Food Safety Authority and the European Center for Disease Prevention and Control recent report, *S. infantis* is the fourth most common serovar detected in humans in the European Union and that is mostly being observed in the turkey and broiler chain. In this report, it has been stated that this serovar has been able to extensively disseminate along the broiler production chain (EFSA, 2017). Indeed it has been suggested that the consumption of contaminated chicken meat is among the most common sources of salmonellosis in humans (Antunes et al., 2016). Furthermore, in Italy, opportunistic pathogen such as *Escherichia coli* isolates producing CTX-M-32, CTX-M-1, and SHV-12 type beta-lactamases were also reported (Giufrè et al., 2012). These strains were retrieved from flocks which had no prior treatment with cephalosporins. It is proposed that the prescription of other antimicrobials such as enrofloxacin and tylosin is responsible for the co-selection of the aforementioned resistant organisms (Bortolaia et al., 2010). Reports on chicken feces (Giufrè et al., 2012), broiler chicken samples, and retail chicken meat (Ghodousi et al., 2016) showed that these latter carried *E. coli* producing CTX-M-grp-1, CTX-M-grp-2, and CTX-M-grp-9 enzymes in Italy. The co-existence of these enzymes with AmpC beta-lactamases was also reported, including CTX-M-1/CMY-2 (Accogli et al., 2013) and CIT-like/CTX-M (Ghodousi et al., 2015) in *E. coli* of poultry origin. CTX-M and AmpC beta-lactamase producers in the Italian poultry belong mostly to the A and B phylogroups with the genes being carried mainly on IncI1 plasmids. In France, the only report from poultry was the detection of two CTX-M-1-producing *E. coli* isolates (Meunier et al., 2006). CTX-M-1 was linked to the insertion sequence IS*Ecp1* (Meunier et al., 2006). This insertion sequence has been previously described as being a potent contributor to the mobilization and insertion of *bla*CTX-M genes (El Salabi et al., 2013). Although no studies described the emergence of ESBL in the Slovenian animal sector, one study reported the presence of CTX-M-1 and SHV-12-producing in Slovenian raw chicken meat samples sold on the Swiss market (Zogg et al., 2016).

In Spain, the Spanish Veterinary Antimicrobial Resistance Surveillance Network (VAV) monitored antimicrobial resistance of *Salmonella enterica* in healthy broilers in 2003–2004: two CTX-M-9 producers were isolated (Riaño et al., 2006). During the same period, ESBL-producing *E. coli* were also detected (Mesa et al., 2006; Moreno et al., 2007). Indeed, it seems that early monitoring systems often targeted resistance in *Salmonella* species, as these are common causative agents of human infections of food of animal origin (Antunes et al., 2016). Thereafter, as bacterial resistance became widely disseminated in all environments (Stoll et al., 2012), researchers began to think of poultry as a reservoir of resistance in enteric organisms. For instance, Egea et al. found that the prevalence of retail poultry meat colonized by CTX-M and/or SHV producing *E. coli* increased from 62.5% in 2007 to 93.3% in 2010 (Egea et al., 2012). During these three years, a significant increase was observed at the level of A0 and D1 phylogroups. Egea et al. suggested that the rise of meat colonization is muli-clonal since only 2 strains from the main phylogroup detected in this study showed genetic relatedness by PFGE typing. Thus, it appears that the diffusion of ESBL producers in retail chicken meat is related rather to successful spread of one or several plasmids carrying the *bla*CTX-M and *bla*SHV genes (Egea et al., 2012). Apart from *E. coli*, ESBL production in the poultry production system in Spain was also detected in *Klebsiella pneumoniae, Enterobacter cloacae, Proteus mirabilis*, and *Serratia fonticola* (Ojer-Usoz et al., 2013). In parallel, CMY-2 is the only AmpC beta-lactamase type reported in *E. coli* originating from chicken in this country (Blanc et al., 2006; Cortés et al., 2010; Solà-Ginés et al., 2015b). Apart from chicken, one study in Spain reported the detection of CTX-M-1, CTX-M-9, CTX-M-14 harboring *E. coli* strains in flies surrounding chicken farms (Solà-Ginés et al., 2015a). For instance, the detection of ESBL producers in flies reflects on one side the contamination status of the farm housing environment; and on the other side, it contributes to the colonization of other broilers with ESBL producing *E. coli* strains (Solà-Ginés et al., 2015a).

In Turkey, the first ESBL production in animals was detected in *K. pneumoniae* and *Klebsiella oxytoca* in 2007–2008 (Gundogan et al., 2011). In 2012–2014, *E. coli* producing CTX-M-1, CTX-M-3, CTX-M-15, CTX-M-8 as well as SHV-5 and SHV-12 were identified in raw chicken meat samples in different areas across the country (Perrin-Guyomard et al., 2016)-(Tekiner and Ozpinar, 2016). The A, D1 and D2 were the most common phylogroups detected. In the same aforementioned study, ESBL was also detected in *E. cloacae, Citrobacter werkmanii*, and *K. pneumoniae* (CTX-M-1) (Tekiner and Ozpinar, 2016). Similarly, CMY-2 type beta-lactamase was detected in *E. coli* (Pehlivanlar Onen et al., 2015) as well as in *E. cloacae* (Tekiner and Ozpinar, 2016). In Lebanon, CTX-M type beta-lactamase followed by CMY AmpC beta-lactamase appear to dominate the Lebanese chicken farms (Dandachi et al., 2018b). MLST typing of CTX-M positive *E. coli* strains revealed the presence of different sequence types across the territory. Furthermore, a significant resistance of ESBL producers toward gentamicin was observed. The spread of ESBL producers in Lebanon could be attributed in part to the co-selective pressure applied by the heavy usage of gentamicin in the veterinary sector as previously reported (Dandachi et al., 2018b). In Israel, only one study showed the presence of CTX-M-producing *E. coli* of A, B, and D phylogroups in liver samples of dead broiler chickens and ready-to-market chicken meat (Qabajah et al., 2014).

Concerning Africa, ESBL was first detected in *E. coli* strains isolated from foods of poultry origin in Tunisia in 2006. These harbored SHV-5, CTX-M-8, CTX-M-14, and CTX-M-1 type beta-lactamases (Jouini et al., 2007). It appears that in this country, *bla*CTX-M-1 and *bla*CMY-2 are the dominant genes responsible for ESBL and AmpC production in *E. coli* isolated from chicken samples (Ben Slama et al., 2010; Ben Sallem et al., 2012). This is in addition to *bla*CTX-M-15, *bla*CTX-M-14 (Maamar et al., 2016), and *bla*CTX-M-9 that were detected in *E. coli* isolated from the fecal samples of dead/diseased chickens (Grami et al., 2014). ESBL genes in Tunisia appear to be located on various plasmids carried by different *E. coli* phylogroups. These include mainly IncI1 followed by IncF and IncFIB (**Table 2**). *bla*CTX-M as well as CMYgenes in Tunisia were found to be also associated to the IS*Ecp1* insertion sequence. Furthermore, apart from the CMY gene, AmpC production in *E. coli* strains in this country was found to be also mediated via mutations in the promoter region of the chromosomal AmpC gene (Ben Slama et al., 2010). In Algeria, CTX-M-like enzymes were detected in *E. coli* (Mezhoud et al., 2015; Chabou et al., 2017) as well as in other species such as ST15 *Salmonella* Heidelberg (Djeffal et al., 2017). In their study, Djeffal et al. reported the detection of the same sequence type “ST15” of *Salmonella* spp isolated from both chicken and human. This emphasizes on the hypothesis that the poultry production system could constitute a potent contributor to the diffusion of multi-drug resistant *Salmonella* in the human population (Djeffal et al., 2017). In parallel, *bla*SHV-12 and CMY-2 genes were detected in *E. coli* strains recovered from slaughtered broilers' intestinal swabs (Belmahdi et al., 2016).

**Figure 1:**
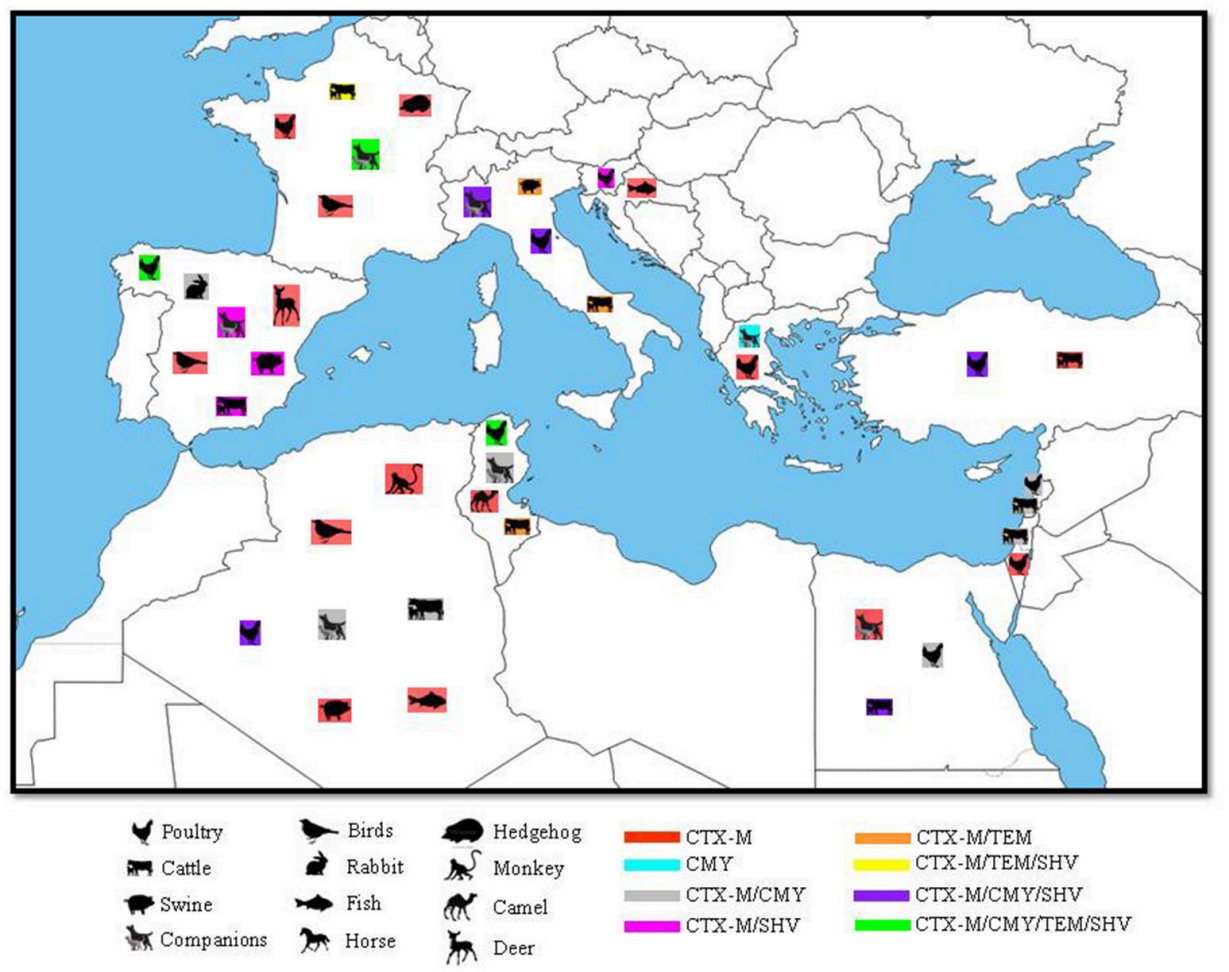
Geographical distribution of ESBLs and their correspondent animal hosts in the Mediterranean Basin. N.B: only SHV and TEM genes confirmed by sequencing as ESBL were included.

In Egypt, *E. coli* producing CTX-M-15 and CMY-2 were initially reported from blood samples from the hearts of septicemic broilers in 2011 (Ahmed and Shimamoto, 2013). CTX-M-15 and CTX-M-14 were further detected in *E. coli, K. pneumoniae, K. oxytoca*, and *Enterobacter spp* isolated from chicken carcasses in the north of Egypt (Abdallah et al., 2015; Ahmed and Shimamoto, 2015). *E. coli* isolates harboring SHV-12 have also been reported in Egypt; although they originated from liver and heart samples of chickens affected with colibacillosis (El-Shazly et al., 2017; Figure 1). Similarly to other countries in the Mediterranean basin, ESBL producers in the Egyptian poultry sector belong mainly to the A and B1 phylogroups with the *bla*CTX-M genes being associated with IS*Ecp1* (**Table 2**).

### Cattle and sheep

Cattle and sheep are essential members of the human food and agricultural system. For humans, cattle and sheep serve as a source of meat and milk. In agriculture, their feces are commonly used as manure for artificial fertilization (Nyberg et al., 2014). As it is now widely recognized that animals' intestines are a normal habitat for wild type and resistant micro-organisms (Nelson et al., 2013), it has been suggested that if resistant bacteria contaminated animal manures are used without prior treatment, there is a potential risk of transmitting this resistance to the surrounding environment and to the human population (Hruby et al., 2016). This transmission may occur through irrigation and drinking water without treatment (Hruby et al., 2016) or through animals grazing on contaminated lands (Bagge et al., 2009).

In France, the first identification of an ESBL producer in cattle dates back to 2004 when *E. coli* strains harboring CTX-M-1 and CTX-M-15 were isolated from cows (Meunier et al., 2006). *E. coli* producing the CTX-M-15 type ESBL were later isolated from the fecal sample of a dead calf (Valat et al., 2012) and from the feces of cattle located in 10 different geographical areas in France (Madec et al., 2012). In the aforementioned study, CTX-M-15 was carried on IncI1 plasmids but also on F31:A4:B1/IncFII and F2:A–:B–/IncFII plasmids which has been extensively reported in humans (Madec et al., 2012). Although CTX-M-15 appears to be dominant in French cattle, other ESBL types were also reported in *E. coli* (Hartmann et al., 2012) and *Klebsiella* species (Dahmen et al., 2013b; Haenni et al., 2014a) such as CTX-M-1, CTX-M-14, CTX-M-9, CTX-M-2, CTX-M-32, CTX-M-57, CTX-M-3 (Dahmen et al., 2013b; Haenni et al., 2014a), and TEM-71(Hartmann et al., 2012). These latter were carried by *E. coli* strains of different sequence types such as ST23, ST58, ST10, ST45, ST88, ST2210, ST2212-ST2215, ST2497, and ST2498 (Table 1); no epidemic clones such as ST101 were detected. Moreover, two studies in France detected AmpC-producing *E. coli* in calves. In both, AmpC beta-lactamase production was suggested as being due to highly conserved mutations in the promotor/attenuator region and to an over-expression of the chromosomal AmpC gene, respectively (Haenni et al., 2014a,c). In sheep, only one study was conducted in France in which one CTX-M-1 *E. fergusonii* and three *K. pneumonia* harboring both *bla*CTX-M-15 and DHA genes were detected (Poirel et al., 2013). The three *K. pneumoniae* were co-resistant to nalidixic acid, sulfonamides, trimethoprim-sulfamethoxazole and tetracycline and belonged to the same sequence type ST274. In Spain, ESBL-producing Gram-negative bacilli were isolated from beef samples collected from different geographical locations (Doi et al., 2010; Ojer-Usoz et al., 2013). In Italy, Stefani et al. reported the isolation of five *Klebsiella ozaenae* harboring CTX-M-1, CTX-M-1/TEM-24 and CTX-M-15 ESBL types from cattle (Stefani et al., 2014).

**Table 1:** Non Beta-lactam resistance in MDR of animal origin vs. antibiotic consumption in the Mediterranean Basin.

**Country**	**Animal host**	**Species (number)**	***bla*gene Type (number)**	**Non beta-lactam Resistance**	**Antibiotic usage**	**References**
Algeria	Poultry	*E. coli* (17)	CTX-M (17)	CMX,NAL,SXT	Unknown	Mezhoud et al., 2015
	Poultry	*E. coli* (16)	CTX-M (2), SHV (14), CMY (4)	AMK, CIP, KAN, NAL, STR, TOB		Belmahdi et al., 2016
	Poultry	*Salmonella spp* (11)	CTX-M (11)	CIP		Djeffal et al., 2017
	Cattle	*A. baumannii* (1)	NDM (1)	CIP		Chaalal et al., 2016; Yaici et al., 2016
	Cattle	*E. coli* (4)	NDM (4), CTX-M (4), CMY (4),			Yaici et al., 2016
	Birds	*E. coli* (11)	CTX-M (11)	CIP, NAL, NEO SXT, TET,		Meguenni et al., 2015
	Birds	*A. baumannii* (4)	OXA (4)			Morakchi et al., 2017
	Dogs	*E. coli* (1)	NDM (1)	FLU, TET		Yousfi et al., 2015
	Dogs	*E. coli* (15)	CTX-M (13), SHV (3)	CIP, GEN, NAL, SUL, SXT, TET, TMP, TOB		Yousfi et al., 2016b
	Dogs	*E. coli* (3)	CTX-M (1), CMY (1), NDM (1), OXA-48 (2)	GEN, CIP, NAL, SXT, TEM, TOB,		Yousfi et al., 2016a
	Cats	*E. coli* (2)	CMY (1), OXA-48 (2)	CIP, GEN, NAL, SXT, TEM, TOB		Yousfi et al., 2016a
	Cats	*E. coli* (5)	CTX-M (5)	CIP, NAL, SUL, SXT, TET, TMP, TOB		Yousfi et al., 2016b
	Fish	*E. coli* (22)	CTX-M (16), TEM (6)	AMK, CIP, CMX, GEN, KAN, NAL, NET, OFX		Brahmi et al., 2016
	Fish	*A. baumannii* (2)	OXA-23 (2)	CIP, GEN, KAN, SXT		Brahmi et al., 2016
	Macaques	*K. pneumoniae* (7)	CTX-M (7)	CIP, FOS, GEN, SXT		Bachiri et al., 2017
	Wild Boars	*E. coli* (30)	CTX-M (30)	AMK, CIP, FOS, GEN, SXT, TET		Bachiri et al., 2017
		*K. pneumoniae* (10)	CTX-M (10)			
Tunisia	Poultry	*E. coli* (13)	CTX-M (12), CMY (1)	CIP, CHL, GEN, NAL, SXT, SUL, STR, TET	Streptomycin, Tetracycline, Sulphonamides, Trimethoprim	Ben Slama et al., 2010; Ben Sallem et al., 2012
	Poultry	*E. coli* (67)	CTX-M (42), CMY (24)	AMK, GEN, NAL, NOR, SXT, TET		Mnif et al., 2012
	Poultry	*E. coli* (16)	CTX-M (16)	NAL, SXT, STR, SUL, TET		Kilani et al., 2015
	Poultry	*E. coli* (7)	CTX-M (7)	NAL, STR, TET, SUL, TMP		Grami et al., 2013
	Poultry	*E. coli* (10)	CTX-M (8), TEM (1), CMY (2)	NAL, SXT, SUL, TET, STR		Ben Sallem et al., 2012
	Poultry	*E. coli* (48)	CTX-M (35), CMY (13)	AMK, CIP, GEN, MIN, NAL, SXT, TET		Maamar et al., 2016
	Poultry	*E. coli* (5)	CTX-M (4), SHV (1)			Jouini et al., 2013
	Cattle	*E. coli* (1)	CTX-M (1)	GEN, TOB, TET		Grami et al., 2014
	Beef	*E. coli* (1)	CTX-M (1)	CIP, NAL, SXT, SUL, TET		Ben Slama et al., 2010
	Beef	*E. coli* (5)	CTX-M (5)	CHL, GEN, STR, SUL, SXT, TET, TOB		Jouini et al., 2013
	Sheep	*E. coli* (3)	CTX-M (5), TEM (1)	CIP, GEN, NAL, SXT, SUL, STR, TET		Ben Slama et al., 2010
	Dogs	*E. coli* (6)	CTX-M (6)	CHL, ENR. GEN, KAN, NAL, NET, SUL, STR, TET, TMP, TOB		Grami et al., 2013
	Dogs	*E. coli* (6)	CTX-M (5), CMY (1)	CIP, NAL, SXT, STR, SUL, TET		Sallem et al., 2013
	Cats	*E. coli* (1)	CTX-M (1)	NAL, STR, SUL, TET, TMP,		Grami et al., 2013
	Cats	*E. coli* (8)	CTX-M (8)	CIP, KAN, NAL, STR, SXT, SUL, TET		Sallem et al., 2013
	Dromedaries	*E. coli* (1)	CTX-M (1)	SUL, TET		Ben Sallem et al., 2012
Egypt	Poultry	*E. coli* (18)	CTX-M (7), CMY (11)	CHL, CIP, KAN, NAL, SPX, STR, SXT, TET	Fluoroquinolones, Tetracyclines, Aminoglycosides, Cefotaxime	Ahmed and Shimamoto, 2013; Dahshan et al., 2015
	Poultry	*E. coli* (9)	CTX-M (2), SHV (1), TEM (1), CMY (1)	CIP, CMX, DOX, GEN, STR		El-Shazly et al., 2017
	Poultry	*K. pneumoniae* (15)	NDM (15), KPC (14), OXA (12)	-		Hamza et al., 2016
	Poultry	*K. pneumoniae* (11) *, K. oxytoca* (1)	NDM (12)			Abdallah et al., 2015
		*E. coli* (8)	CTX-M (8)			
		*K. pneumoniae* (40)	CTX-M (40)			
		*K. oxytoca* (2)	CTX-M (2)			
		*Enterobacter spp* (9)	CTX-M (9)			
	Cattle	*E. coli* (112)	CTX-M (106), OXA (6)	FOS, FLU, CMX, CHL, MLS, TET,	Tetracycline, quinolones	Braun et al., 2016
	Cattle	*E. coli* (8)	CTX-M (2), SHV (5), CMY (1)	NAL, SXT, STR, TET		Ahmed et al., 2009
	Beef	*E. coli* (4)	CTX-M (1), SHV (1), CMY (2)	CHL, CIP, GEN, KAN, NAL, SPX, STR, SXT, TET	Fluoroquinolones	Ahmed and Shimamoto, 2015
	Cats	*E. coli* (5)	CTX-M (5)			Abdel-Moein and Samir, 2014
	Dogs	*E. coli* (11)	CTX-M (11)			Abdel-Moein and Samir, 2014
		*K. pneumoniae* (3)	CTX-M (3)			
		*P. mirabilis* (1)	CTX-M (1)			
Palestine	Cattle	*E. coli* (287)	CTX-M (287)	SXT, STR, TET	Chlortetracycline, doxycycline, Norfloxacin, Cephalexin, Ceftiofur, Sulfa agents, Gentamicin, Monensin	Adler et al., 2015
		*K. pneumoniae* (4)	SHV (4)	CHL, CIP, GEN		
	Poultry	*E. coli* (9)	CTX-M (9)			Qabajah et al., 2014
Lebanon	Poultry	*E. coli* (217), *K. pneumoniae* (8), *P. mirabilis* (3), *E. albertii* (2), *E. fergusonii* (1), *E. cloacae* (3),	CTX-M, CMY	CIP, GEN, SXT	Gentamicin, Tetracyclines	Dandachi et al., 2018a
	Cattle	*E. coli* (27)	CTX-M (27)	CHL, ENR, GEN, KAN, NAL, STR, SUL, TET, TMP	Penicillin G - Streptomycin, Ampicillin, Amoxicillin Oxytetracycline, Gentamicin,	Gundogan et al., 2011; Diab et al., 2016
	Fowl	*A. baumannii* (1)	OXA-48 (1)	AMK, GEN, TOB	Unknown	Al Bayssari et al., 2015b
	Horse	*A. baumannii* (1)	OXA-143 (1)			Rafei et al., 2015
	Rabbit	*A. pitii* (1)	OXA-24 (1)			
Turkey	Poultry		CTX-M (60), SHV (4), CMY (18)	CHL, KAN, NAL, STR, SUL, TET, TMP	Tetracycline, Quinolones	Politi et al., 2005; Pehlivanlar Onen et al., 2015
	Cattle	*E. coli* (3)	CTX-M (2), CMY (1)	NAL, SXT, STR, TET		
	Poultry	*E. coli* (15)	CTX-M (15)			Tekiner and Ozpinar, 2016
	Cattle	*E. coli* (19)	CTX-M (19)			
Croatia	Mussel	*Aeromonas. Caviae* (25)	CTX-M (11), SHV (11), FOX (3)		Tetracycline, Amphenicol, Penicillins, Sulfonamides, Trimethoprim, Fluoroquinolones, Aminoglycosides, Polymixins	Maravić et al., 2013; EMA/ESVAC, 2014
		*A. Hydrophila* (8)	CTX-M (8), SHV (2)			
Greece	Poultry	*Salmonella enteric* (2)	CTX-M (2)	CHL, KAN, STR, SUL, TMP, TET	Unknown	Politi et al., 2005
	Dogs	*E. coli* (8)	CMY (8)	FLU		Vingopoulou et al., 2014
Slovenia	Poultry	*E. coli* (6)	CTX-M (2), SHV (4)	GEN, NAL, STR, SUL	Ceftiofur	Chiaretto et al., 2008
Italy	Poultry, Cattle, Swine				Tetracyclines, Amphenicol, Penicillins, 3rd/4th Cephalosporins, Sulfonamides, Trimethoprim, Macrolides, Lincosamides, Fluoroquinolones, Aminoglycosides, Polymixins, Pleuromutilins, Tylosin, Flumequine,	
	Poultry	*E. coli* (8)	CTX-M (7), SHV (1),	CIP		Giufrè et al., 2012
	Poultry	*E. coli* (60)	CTX-M (45), CIT-like (15)	CIP, GEN, SXT, TET		Ghodousi et al., 2015
	Poultry	*E. coli* (67)	CTX-M (24), SHV (43)	CIP, NAL, SUL, TMP, TET		Bortolaia et al., 2010
	Poultry	*Salmonella spp* (12)	SHV (12)	GENT, NAL, SUL, STR, TET		Chiaretto et al., 2008
	Poultry	*Salmonella infantis* (30)	CTX-M (30)	CIP, NAL, SUL, TMP, TET		Franco et al., 2015
	Swine	*Salmonella infantis* (2)	CTX-M (2)			
	Cattle	*K. ozaenae* (5)	CTX-M (5), TEM (1)			Stefani et al., 2014
	Swine	*E. coli* (15)	CTX-M (10), TEM (7)			
	Dogs	*K. oxytoca* (2)	SHV (2), DHA (2)	CIP, GEN, KAN, STR, SUL, TET, TMP		Donati et al., 2014
		*K. pneumoniae* (11)	CTX-M (11), SHV (5), DHA (1)	CIP, GEN, KAN, NAL, TET, TMP		
	Dogs	*K. pneumoniae* (1)	CTX-M (1), SHV (1)	CIP, LEV		Bogaerts et al., 2015
		*E. coli* (1)	CMY (1)	CIP, LEV		
	Cats	*K. oxytoca* (2)	CTX-M (2)	CIP, SUL, TMP, TET		Donati et al., 2014
		*K. pneumoniae* (4)	CTX-M (2), SHV (2), DHA (1), CMY (1)	CIP, KAN, NAL, SUL, TET, TMP		
	Cats	*E. coli* (7)	CTX-M (7), CMY (2)	CHL, ENR, GEN, NAL, NIT, SPX, STR, SUL, TET, TMP.		Nebbia et al., 2014
France	Poultry, Cattle, Swine				Tetracycline, Amphenicol, Penicillins, 1st/2nd/3rd/4th Cephalosporins, Sulfonamides, Trimethoprim, Macrolides, Lincosamides, Fluoroquinolones, Aminoglycosides, Polymixins, Pleuromutilins	EMA/ESVAC, 2014
	Cattle	*E. coli* (26)	CTX-M (21), TEM (5)	CHL, GENT, SXT		Hartmann et al., 2012
	Cattle	*E. coli* (3)	CTX-M (3)	CHL, ENR, FFC, GEN, KAN, NAL, STR, SUL, TET, TMP		Meunier et al., 2006
	Cattle	*A. baumannii* (9)	OXA-23 (9)	FOS, KAN, TET		Poirel et al., 2012
	Cattle	*E. coli* (9)	CTX-M (9)	CHL, ENR, GEN, KAN, NAL, NET, OFX, STR, SUL, TET, TOB, TMP		Madec et al., 2012
	Cattle	*E. coli* (5)	CTX-M (5)	APR, CHL, ENR, GEN, KAN, NAL, NET, OFX, STR, SUL, TET, TOB, TMP		Dahmen et al., 2013b
		*K. pneumoniae* (1)	CTX-M (1)			
	Sheep	*K. pneumoniae* (3)	CTX-M (3), DHA (3)	NAL, SUL, SXT, TET		Poirel et al., 2013
		*E. fergusonii*	CTX-M (1)			
	Veal calves	*E. coli* (147)	CTX-M (147)	APR, CHL, ENR, FFC, GEN, KAN, NAL, NET, SUL, STR, TET, TOB, TMP		Haenni et al., 2014a
		*K. pneumoniae* (3)	CTX-M (2), SHV (1)	FLU, SUL, STR, TET, TMP		
	Swine	*E. coli* (3)	CTX-M (3)	CHL, NAL, STR, SUL, TET, TMP		Meunier et al., 2006
	Dog	*E. cloacae* (11)	CTX-M (10), SHV (1)	FLU, GEN, KAN, QUI, TET, SUL, STR, TMP		Haenni et al., 2016c
	Dog	*E. coli* (47)	CTX-M (47), CMY (24)	CHL, GEN, KAN, STR, TOB ENR, FFC, NAL, NET, OFX, SUL, TET, TMP		Haenni et al., 2014a
	Dog	*E. coli* (9)	CTX-M (8), TEM (1)	GEN, SUL, TET		Poirel et al., 2013
		*K. pneumoniae* (8)	CTX-M (8), DHA (1)	GEN, NAL, SUL, SXT, TET		
		*K. oxytoca* (2)	CTX-M (2)			
	Dog	*P. mirabilis* (14)	CTX-M (1), CMY (7), DHA (2), VEB (6)	APR, CHL, ENR, GEN, KAN, NAL, NET, STR, SUL, TOB, TMP		Schultz et al., 2017
	Dog	*A. baumannii* (2)	OXA-23 (2)	CIP, SXT		Hérivaux et al., 2016
	Dog	*E. coli* (3)	CMY (2), OXA-48 (1)	GEN, NAL		Melo et al., 2017
	Cat	*A. baumannii* (1)	OXA-23 (1)	GEN, NAL, SUL, STR, TET		Ewers et al., 2016
	Cat	*K. pneumoniae* (3)	CTX-M (3), DHA (3)	NAL, SUL, SXT, TET	Unknown	Poirel et al., 2013
		*E. coli* (3)	CTX-M (3)	GEN, SUL, TET	Unknown	
	Cat	*P. mirabilis* (1)	CMY (1)	ENR, NAL, SUL, TMP		Schultz et al., 2017
		*P. rettgeri* (1)	CTX-M (1)	ENR, NAL, SUL, TMP		
	Cat	*E. coli* (2)	CTX-M (2)	STR, TMP		Melo et al., 2017
	Cat	*E. cloacae* (11)	CTX-M (10), SHV (1)	FLU, GEN, KAN, QUI, SUL, STR, TET, TMP		Haenni et al., 2016c
	Companions	*E. coli* (19)	CTX-M (19)	CIP, NAL, SUL, STR, TET		Dahmen et al., 2013a
	Hedgehog	*E. coli* (1)	CTX-M (1), DHA (1)	NAL, SUL, SXT, TET	Unknown	Poirel et al., 2013
	Tawny Owl	*E. coli* (1)	CTX-M (1)			
	Domestic goose	*E. coli* (1)	CTX-M (1)			
	Rock Pigeon	*E. coli* (1)	CTX-M (1)			
	Horse	*E. cloacae* (14)	CTX-M (8), SHV (6)	FLU, GEN, KAN, QUI, SUL, STR, TET, TMP		Haenni et al., 2016c
	Horse	*P. mirabilis* (14)	VEB (2)	ENR, CHL, KAN, NAL, NET, SUL, STR, TOB, TMP	Unknown	Schultz et al., 2017
Spain	Poultry, Cattle, Swine				Tetracycline, Amphenicol, Penicillins, 3rd/4th Cephalosporins, Sulfonamides, Trimethoprim, Macrolides, Lincosamides, Fluoroquinolones, Quinolones, Aminoglycosides, Polymixins, Pleuromutilins	Abreu et al., 2014; EMA/ESVAC, 2014
	Poultry	*E. coli* (64)	CTX-M (44), SHV (6), TEM (2), CMY (13)	CHL, CIP, FUR, GEN, KAN, NAL, SUL, SXT, TET, TOB, TMP		Blanc et al., 2006
	Poultry	*S. enterica* (2)	CTX-M (1), SHV (1)	NAL, SXT, STR, SUL, TET,		Riaño et al., 2006
	Poultry	*E. coli* (116)	CTX-M (116)	CIP, NAL, SXT		Abreu et al., 2014
	Poultry	*E. coli* (11)	CTX-M (6), SHV (2), CMY (2)	CHL, CIP, FFC, GEN, KAN, NAL, STR, SUL, TET, TMP		Solà-Ginés et al., 2015b
	Poultry	*E. coli* (50)	CTX-M (40), CMY (10)	NAL		Cortés et al., 2010
	Poultry	*E. coli* (62)	CTX-M (20), SHV (42)	CIP, NAL		Egea et al., 2012
	Swine	*E. coli* (20)	CTX-M (20)			Solà-Ginés et al., 2015b
	Swine	*S. enteric* (1)	SHV (1)	SUL, STR, TET		Riaño et al., 2006
	Swine	*E. coli* (39)	CTX-M (27), SHV(12)	CIP, CHL, FUR, GEN, KAN, NAL, SUL, SXT, TET, TMP, TOB		Blanc et al., 2006
	Swine	*E. coli* (20)	CTX-M (8), SHV (12)	APR, CIP, GEN, NAL, STR, SUL, TET, TMP		Escudero et al., 2010
	Dog	*E. coli* (1)	SHV (1)	CHL, CIP, NAL, SUL, TET, TMP		Teshager et al., 2000
	Dog	*E. coli* (1)	CMY (1)			Bogaerts et al., 2015
		*P. mirabilis* (2)	CMY (2)	DOX, MIN		
	Dog	*K. pneumoniae* (2)	CTX-M (1), VIM (1), DHA (1)			González-Torralba et al., 2016
		*E. cloacae* (1)	SHV (1)			
	Deer	*E. coli* (1)	CTX-M (1)	CIP, CHL, NAL, SXT, TET	Unknown	Alonso et al., 2016
	Rabbit	*E. coli* (1)	CMY (1)		Unknown	Blanc et al., 2006
		*E. cloacae* (3)	CTX-M (3)			

^*^APR, refers to apramycin; AMK, amikacin; CIP, ciprofloxacin; CHL, chloramphenicol; CMX, co-trimoxazole; DOX, doxycycline; ENR, enrofloxacin; FFC, florfenicole; FLU, fluoroquinolones; FOS, fosfomycin; FUR, furazolidone; GEN, gentamicin; KAN, kanamycin; LEV, levofloxacin; MIN, minocycline; MLS, Macrolides; NAL, nalidixic acid; NET, netilmicin; NIT, nitrofurantoin; NOR, norfloxacin; OFX, oxofloxacin; QUI, quinolones; SPX, spectinomycin; SXT, trimethoprim-sulfamethoxazole; TEM, temocillin; TET, tetracycline; TMP, trimethoprim; TOB, tobramycin.

In Turkey, a study conducted in 2007–2008, showed the presence of ESBL-producing *K. pneumoniae* and *K. oxytoca* in raw calf meat (Gundogan et al., 2011). Later on, CTX-M-3 and CTX-M-15 harboring *E. coli* were isolated from beef samples sold in a market in the south of Turkey (Conen et al., 2015). Recently, a study conducted by Tekiner et al. reported the isolation of ESBL-producing *E. coli, E. cloacae*, and *Citrobacter brakii* from raw cows' milk collected from different cities of Turkey. In these areas, CTX-M-1 was dominant (Tekiner and Ozpinar, 2016). In Lebanon the situation differs, in that unlike Turkey but similarly to other Mediterranean countries, *bla*CTX-M-15, *bla*SHV-12, and *bla*CTX-M-14 are the dominant ESBL genes prevailing in *E. coli* in the Lebanese cattle (Diab et al., 2016). In this latter study, various sequence types were detected. Of special interest is the detection of ST10. ST10 was heavily reported in the literature as being shared between animal and human isolates all over the world: Chile (Hernandez et al., 2013), Denmark (Huijbers et al., 2014), Vietnam (Nguyen et al., 2015), Germany (Belmar Campos et al., 2014). Indeed, it has been suggested that ST10 became associated with the production and dissemination not only of CTX-M-type ESBLs but also of *mcr-1* in animals, humans and environment (Monte et al., 2017). In Israel, Adler et al. reported the identification of CTX-M-1/CTX-M-9 and SHV-12 beta-lactamase producing *E. coli* and *K. pneumoniae* strains respectively, which were isolated from cattle farms situated in the main farming locations across the country (Adler et al., 2015).

In Egypt, SHV-12 (Ahmed et al., 2009) in addition to CTX-M-1/15 and CTX-M-9 were detected in *E. coli* strains isolated from cattle (Braun et al., 2016). On study targeting raw milk samples reported the detection of SHV-12 /CTX-M-3, in addition to CMY-2-producing *E. coli* strains (Ahmed and Shimamoto, 2015). In Tunisia, *E. coli* strains producing CTX-M-1 and TEM-20 were isolated from beef and sheep situated in different areas across the country (Jouini et al., 2007; Ben Slama et al., 2010). Furthermore, *bla*CTX-M-15 was detected in an ST10 *E. coli* isolate recovered from the milk sample of cattle affected with mastitis (Grami et al., 2014). Similarly, In Algeria, Yaici et al. reported the detection of four ST1284 *E. coli* strains carrying CTX-M-15, CMY-42, and NDM-5 in raw milk samples (Yaici et al., 2016).

### Swine

Meat from pigs is used by humans for consumption and their feces are used as manure for land fertilization. Studies have shown that antibiotics are usually detected in higher concentrations in pig manures compared to that of other farm animals (Hou et al., 2015). This finding reflects high and uncontrolled antimicrobial usage in swine farms (Woolhouse et al., 2015). Heavy antibiotic usage creates a selective pressure that contributes to the emergence and spread of bacterial resistance; in this regard, pigs are suggested as a potential source of resistant bacteria.

Reports concerning the prevalence of ESBL of swine origin in the Mediterranean area are very scarce with the majority being reported from Spain where a *bla*SHV-12 positive *Salmonella enterica* was isolated in the early 2000s (Riaño et al., 2006). Furthermore, CTX-M-grp-9 (Doi et al., 2010; Ojer-Usoz et al., 2013), SHV-5 and CTX-M-grp-1 carried by A phylogroup *E. coli* strains and SHV-12 carried by B1 *E. coli* and *bla*SHV-5 were detected (Blanc et al., 2006; Cortés et al., 2010). One study conducted in 13 different Spanish provinces found seven AmpC-producing *E. coli*. In these cases, AmpC production was due to a mutation in the promoter region of the chromosomal AmpC gene (Escudero et al., 2010). In Italy, TEM-52, CTX-M-1, CTX-M-15, and CTX-M-1/TEM-201 carrying *E. coli* were reported in pigs (Stefani et al., 2014). Franco et al. reported also the presence of *Salmonella* infantis carrying CTX-M-1 in swine (Franco et al., 2015). In France, only one study conducted at the beginning of the Twenty-first century reported the detection of CTX-M-1-producing *E. coli* strains in pigs (Meunier et al., 2006). Similarly to what is widely observed in the Mediterranean basin, the CTX-M-1 was associated with the insertion sequence IS*Ecp1*(Meunier et al., 2006). In Algeria, CTX-M-15 harboring *E. coli* and *K. pneumoniae* strains were isolated in 2014 from wild boars (Bachiri et al., 2017). MLST typing showed the *K. pneumoniae* belongs to the ST584 while on the other hand several sequence types (ST617, ST131, ST648, ST405, ST1431, ST1421, ST69, ST226) were observed among *E. coli* strains (Bachiri et al., 2017). The aforementioned study was the only one to investigate the epidemiology of ESBL-producing Gram-negative bacilli in the African and Asian countries lining the Mediterranean Sea.

### Companion animals

Unlike food producing animals, companion animals are not used as consumption source of human food, nor are their feces used as manure for land fertilization. Instead, these animals are kept for the individual's protection, entertainment and company. The number of companion animals has significantly increased in modern society in recent decades (Pomba et al., 2017). Despite regular close contact with people, little attention has been given to the prevalence of antimicrobial resistance in these animals (Scott Weese, 2008). The close contact between companion animals such as dogs, cats, and horses and their owners makes the transmission of resistant organisms more likely to occur (Dierikx et al., 2012). As such, it is essential to investigate the prevalence of resistant bacteria in companion animals as well as to identify the possible risk factors for the transmission of resistant organisms to humans (Rubin and Pitout, 2014).

In the Mediterranean basin, the first detection of ESBL in companion animals was in Spain where an *E. coli* harboring SHV-12 was isolated from a dog with a urinary tract infection (Teshager et al., 2000). Subsequently, between 2008 and 2010, three strains carrying CMY-2 (one ST2171 *E. coli* and two *P. mirabilis*) were recovered from dogs infected with respiratory, urinary tract and skin and soft tissue infections, respectively (Bogaerts et al., 2015). In all three strains, the CMY-2 genes were associated with the IS*Ecp1*. More recently, one *K. pneumoniae* and one *E. cloacae* producing CTX-M-15/DHA and SHV-12, respectively, were isolated from the fecal swabs of healthy dogs in this same country (González-Torralba et al., 2016).

In Italy, a study conducted by Donati et al. on 1,555 dog samples of clinical cases and necropsy specimens with suspicious bacterial infections, between the center and the north of Italy found two *K. oxytoca* harboring SHV-12/DHA-1 and 11 *K. pneumoniae* carrying the following genes: *bla*CTX-M-15 (six strains), *bla*CTX-M-15/DHA-1, *bla*CTX-M-15/SHV-28, *bla*CTX-M-1/SHV-28, and *bla*CTX-M-1 (Donati et al., 2014). In this same study, 429 cats' samples were also investigated revealing the presence two *K. oxytoca* producing CTX-M-9 and four *K. pneumoniae* producing CTX-M-15 (two isolates), CTX-M-15/ DHA-1 and SHV-28/CMY-2 beta-lactamases (Donati et al., 2014). The beta-lactamase and AmpC genes in *K. oxytoca* strains isolated from dogs and cats were located on different plasmid types: IncL/M versus IncHI2 respectively. This is unlike the *K. pneumoniae* strains where the *bla*CTX-M-15 was localized on the same plasmid IncR and both strains in dogs and cats shared the same ST340. ST15 and ST101 were also common between dogs and cats in this study. ST15 and ST101 are among the most international clones carrying ESBL as well as carbapenemase genes which became highly detected recently worldwide (Donati et al., 2014). Another study conducted reported the detection of CTX-M-1-producing *K. pneumoniae* was further reported from a dog with urinary tract infection and an *E. coli* carrying the CMY-2 type beta-lactamase associated to IS*Ecp1* also in a diseased cat with a urinary tract infection (Bogaerts et al., 2015). Infections in pets with *E. coli* strains carrying CTX-M-14 (three isolates), CTX-M-15, CTX-M-1, and CTX-M-14/CMY-2 (two isolates) were also reported in Italy (Nebbia et al., 2014). The strains also showed different sequence types and phylogroups (A “ST3848, ST3847,” B2 “ST131, ST155, ST555, ST4181,” B1 “ST602”) emphasizing that apparently the dissemination of ESBL and AmpC beta-lactamase producers is most likely due to the successful spread of various plasmids carrying these resistance genes (Nebbia et al., 2014).

In France, the highest number of studies addressing the prevalence of extended-spectrum-cephalosporin resistance in companion animals in the Mediterranean was conducted. In dogs, CTX-M-grp 1 (CTX-M-1, CTX-M-15, CTX-M-3, CTX-M-32) and CTX-M-grp 9 in addition to CMY-2 and TEM-52 prevail in *E. coli* (Dahmen et al., 2013a; Poirel et al., 2013; Haenni et al., 2014b; Bogaerts et al., 2015; Melo et al., 2017). These genes were mostly carried on IncI1, IncFII, and IncHI2 plasmid types and were harbored by strains of different sequence types and phylogroups. Furthermore, *K. pneumoniae* isolated from dogs showed to produce the CTX-M-15, CTX-M-32, SHV-12, and DHA-1 have been reported (Poirel et al., 2013; Haenni et al., 2014b). In parallel, *P. mirabilis* showed to produce CMY-2, DHA-16, VEB-6, and CTX-M-15 have been described (Schultz et al., 2017) and *E. cloacae* the CTX-M-15, CTX-M-14, CTX-M-3, and SHV-12 have been identified (Haenni et al., 2016c). In addition, CTX-M-15 and CMY-2 were also decribed in *K. oxytoca* and *Salmonella enterica*, respectively isolated from dogs in this same country (Poirel et al., 2013; Haenni et al., 2014b). On the other hand, in cats, the following distribution was observed: in *E. coli* (CTX-M-1, CTX-M-15, CTX-M-32, CTX-M-3, CTX-M-14) (Poirel et al., 2013; Melo et al., 2017), in *K. pneumoniae* (CTX-M-15/DHA) (Poirel et al., 2013), in *E. cloacae* (CTX-M-15, SHV-12) (Haenni et al., 2016c), in *P. mirabilis* (CMY-2) and in *Proteus rettgeri* (CTX-M-1) (Schultz et al., 2017). The dissemination of extended-spectrum-cephalosporin resistance in companion animals in France necessitates studies addressing the risk factors responsible for the acquisition of these strains in pets as well as novel approaches to control the spread of resistance in these animals. Furthermore, the contribution of the pet animals to the spread of resistance in the common population in France should be also investigated. Moreover, France is the only Mediterranean country in which studies reporting ESBL and/or AmpC-producing bacteria in horses are available. Between 2010 and 2013, *E. cloacae* harboring CTX-M-15, CTX-M-1, and SHV-12 were isolated from clinical samples of horses. These genes were located on IncHI2 and IncP plasmids and were harbored by strains of various sequence types such as ST127, ST372, ST145, ST114, ST135, ST118, ST268, ST107 (Haenni et al., 2016c). Later on, VEB-6 carrying *P. mirabilis* were isolated from healthy horses (Schultz et al., 2017). In Greece, CMY-2 carried on IncI1 plasmid and harbored by ST212 *E. coli* strains were isolated from diseased canines in 2011 (Vingopoulou et al., 2014). More recently, a study conducted in Greek households revealed the detection of extended-spectrum-cephalosporin-resistant *E. coli* isolates. The strains presented with different sequence types including the human pandemic ST131 clone which suggests a possible from humans to animals and vice-versa (Liakopoulos et al., 2018).

In Egypt, CTX-M beta-lactamases have been detected in *E. coli* recovered from cats' rectal swabs. In this same study, CTX-M-producing *E. coli, K. pneumonia*, and *P. mirabilis* were isolated from dogs (Abdel-Moein and Samir, 2014). In Algeria, only one study reported the detection of *E. coli* strains carrying *bla*CTX-M-1, *bla*CTX-M-15 in cats and *bla*CTX-M-1, *bla*CTX-M-15, *bla*SHV-12 in dogs (Yousfi et al., 2016b). In Tunisia, CTX-M-1 carrying *E. coli* were isolated from cats; while from dogs CTX-M-1, CTX-M-15, and CMY-2-producing *E. coli* were detected (Grami et al., 2013; Sallem et al., 2013). CTX-M-1 was mostly carried on IncI1 plasmid whereas CTX-M-15 on IncFII (Grami et al., 2013). The *bla*CTX-M-1 and CMY-2 genes were also found associated with the IS*Ecp1*. Indeed it appears that the insertion sequence IS*Ecp1* might be also responsible for the dissemination of CMY-2 AmpC genes apart from the *bla*CTX-M ones.

### Wild birds and domestic animals

Besides companion and food producing animals, scattered reports exist on the isolation of ESBL from domestic animals such as wild birds and dromedaries in the Mediterranean. For instance, CTX-M-producing *E. coli* was isolated from wild birds in Algeria (Meguenni et al., 2015), Turkey (Yilmaz and Guvensen, 2016), *bla*CTX-M-1 in addition to *bla*CTX-M-15 carrying *E. cloacae* in France (Bonnedahl et al., 2009). Furthermore, in France, CTX-M-1 and CTX-M-15 were detected in ST93, ST124, and ST10 *E. coli* strains recovered from tawny owls/rock pigeons and domestic geese, respectively. In addition, a CTX-M-15/DHA-producing ST274 *K. pneumoniae* was isolated from a hedgehog living in the same city (Poirel et al., 2013). Rooks carrying CTX-M-14 type ESBL in *E. coli* have been described in Italy and Spain (Jamborova et al., 2015). Furthermore, in Spain, *E. coli* and *K. pneumoniae* harboring CTX-M-14, CTX-M-1, CTX-M-32, CTX-M-9, CTX-M-15, CTX-M-14b, CTX-M-3, and CTX-M-8 were recovered from the fecal samples of gulls (Stedt et al., 2015). In rabbits, CMY-2-producing *E. coli* and CTX-M-14, CTX-M-9-producing *E. cloacae* were isolated (Blanc et al., 2006; Mesa et al., 2006). More recently, *bla*CTX-M-1 was identified in *E. coli* isolated from the fecal sample of a deer living in the Los Alcornocales natural park in southern Spain (Alonso et al., 2016). In Algeria, *bla*CTX-M-15 and *bla*CTX-M-9 genes were detected in *E. coli* isolated from the gut and gills of fish caught in the Mediterranean across Bejaia city (Brahmi et al., 2016). In this study, it has been suggested that the presence of beta-lactamase producers is due to contamination of the fish from river water and the rising amount of untreated waste that is released into the Mediterranean Sea from the agricultural as well as the industrial operations (Brahmi et al., 2016). These findings emphasizes on the importance of the natural environment in the dissemination of resistance from humans to animals and vice versa. Furthermore, Bachiri et al. also reported the detection of CTX-M-15-producing ST584 *K. pneumoniae* in Barbary macaques situated in national parks in the north of Algeria (Bachiri et al., 2017). In both Tunisia and Egypt, CTX-M beta-lactamases were detected in *E. coli* and *Pseudomonas aeruginosa* recovered from dromedaries and camels, respectively (Ben Sallem et al., 2012; Elhariri et al., 2017). In Croatia, the only study investigating the prevalence of ESBL in animals was conducted in 2009–2010 in mussels caught in the Adriatic Sea. In this study, 18 *Aeromonas* species carrying SHV-12, CTX-M-15, FOX-2, and PER-1 were identified (Maravić et al., 2013).

### Prevalence of carbapenemase producers in livestock and domestic animals

Carbapenems are beta-lactam antibiotics often considered as the last resort antimicrobial agent against multi-drug resistant organisms (Temkin et al., 2014). Carbapenems are active against ESBL and AmpC-producing Gram negative bacilli. Due to the wide dissemination of multi-drug resistant organisms, these antimicrobials recently became heavily used in human medicine. As a result, the emergence of carbapenem resistance has accelerated and it is now a normal phenomenon encountered in hospital settings and, to a lesser extent, community settings. The production of hydrolyzing enzymes called “carbapenemases” is one of the mechanisms by which carbapenem resistance is mediated in Gram negative bacilli. These include (a) class A carbapenemases (KPC, GES, SME, IMI, NMC-A), (b) class B metallo beta-lactamases “MBL” (NDM, VIM, IMP and TMB), and (c) class D oxacillinases (Martínez-Martínez and Gonzalez-Lopez, 2014).

**Figure 2:**
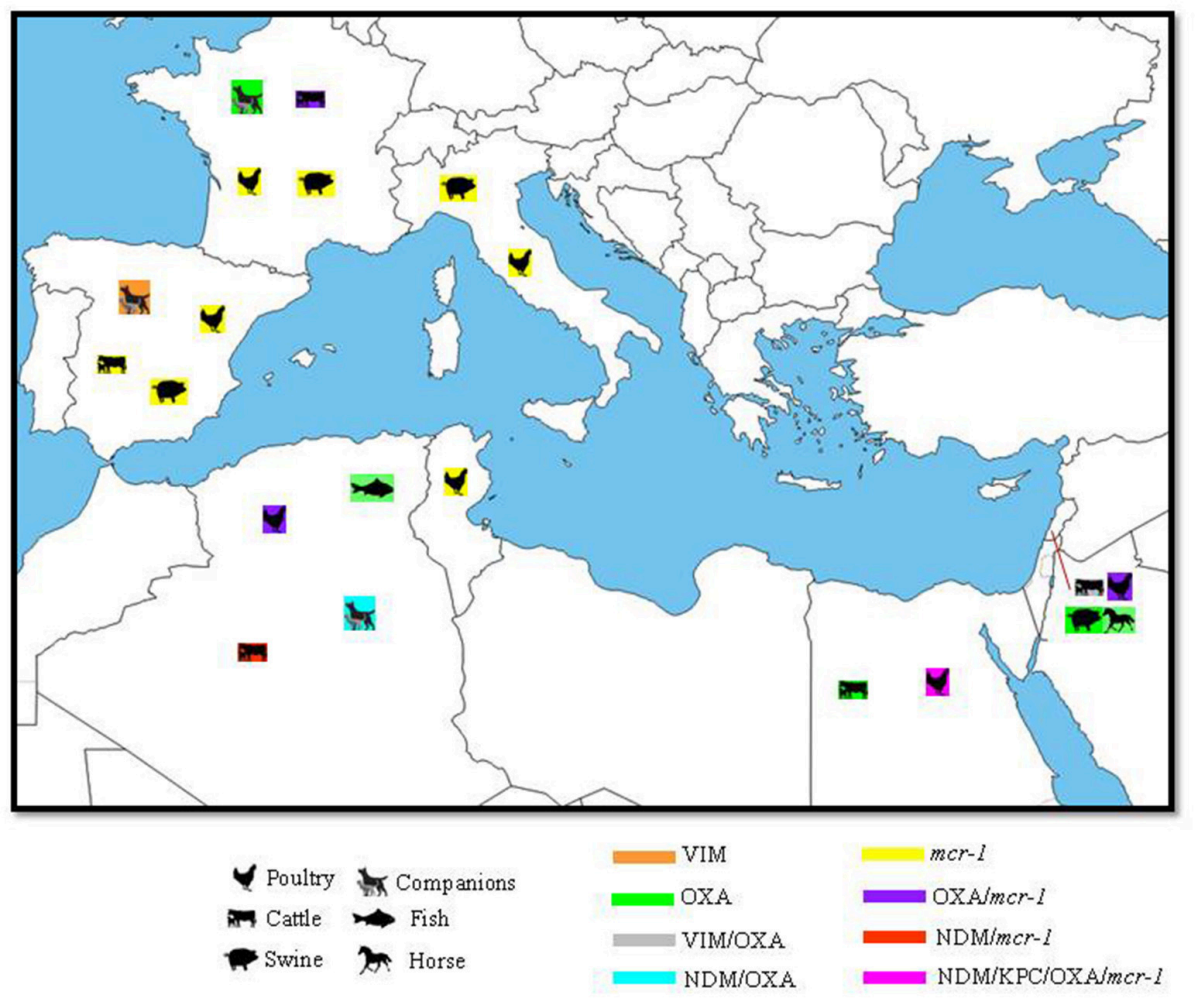
Geographical distribution of carbapenemases and *mcr* colistin resistance gene with their hosts in the Mediterranean. N.B: only OXA genes confirmed by sequencing as carbapenemases were included.

In the Mediterranean basin, in Egypt, OXA-48 and OXA-181 carbapenemases were detected in *E. coli* strains recovered from dairy cattle farms (Braun et al., 2016). In the poultry production system, one study reported the isolation of *K. pneumonia* and *K. oxytoca* harboring NDM metallo beta-lactamases (Abdallah et al., 2015). Another study described the identification of *K. pneumoniae* carrying OXA-48, NDM and KPC type carbapenemases. Isolated strains were recovered from the liver, lungs, and trachea of broiler chicken (Hamza et al., 2016). In Algeria, NDM-1 and NDM-5 were observed, respectively, in ST85 *Acinetobacter baumannii* and ST1284 *E. coli* originating from raw milk in the west and north of the country (Chaalal et al., 2016; Yaici et al., 2016). In *E. coli*, NDM-5 was located on an IncX3 plasmid (Yaici et al., 2016). In broilers, OXA-58 was identified (Chabou et al., 2017) while in pigeons, in addition to OXA-58 and OXA-23 were detected (Morakchi et al., 2017). In terms of companion animals, NDM-5 and OXA-48-producing *E. coli* were reported from healthy dogs Algeria (Yousfi et al., 2015, 2016a). The NDM-5 was harbored by an *E. coli* strain having the same sequence type ST1284 previously described in cattle (Yousfi et al., 2015; Yaici et al., 2016). OXA-48 was further detected in healthy and diseased cats in the same city (Yousfi et al., 2016a). Furthermore, in this same country, two *A. baumannii* producing OXA-23 were isolated from fish (Brahmi et al., 2016). In Lebanon, *A. baumannii* with different sequence types (ST294, ST491, ST492, ST493) were detected in a horse's mouth carrying OXA-143 (Rafei et al., 2015), and in pigs and cattle carrying OXA-23(Al Bayssari et al., 2015a). Furthermore, in cattle, a VIM-2-producing *P. aeruginosa* was isolated (Al Bayssari et al., 2015a). In fowl, Bayssari et al. reported the detection of OXA-23 and OXA-58 harboring *A. baumannii* and OXA-48-producing *E. coli* as well as VIM-2 producing *P. aeruginosa* (Al Bayssari et al., 2015b). VIM-2 producers in fowl and cattle were of different sequence types suggesting the presence of plasmid that is mediating the spread of this resistance gene. In France, OXA-23-producing *Acinetobacter* species were described in cows and dogs (Poirel et al., 2012; Hérivaux et al., 2016). Melo et al. reported the detection of OXA-48 located on an IncL plasmid and carried by an ST372 *E. coli* strain from dogs in France (Melo et al., 2017). In contrast, in Spain, only one study reported the isolation of a VIM-1-producing ST2090 *K. pneumoniae* from a dog's rectal swab (González-Torralba et al., 2016; Figure 2).

### Clonal relationship of beta-lactamase producers and plasmid types of beta-lactamase genes isolated from all animal sources

The different phylogroups and sequence types of beta-lactamase and *mcr-1* positive strains as well as the type of plasmids carrying ESBL, AmpC, carbapenemase, and *mcr-1* genes detected in all animal sources in the Mediterranean region are summarized in Table 2. In this area of the world, it appears that multi-drug resistance in the veterinary sector is mediated by the spread of different phylogroups and sequence types with the main ones being A, B, and D phylogroups (Table 2). The detection of ST10 in CTX-M producers in poultry, cattle, pets, and domestic animals in Algeria, Tunisia, Lebanon, and France is of special interest. ST10 was often described in the literature as being common to ESBL *E. coli* strains of human and avian origin worldwide such as in Germany (Belmar Campos et al., 2014), Denmark (Huijbers et al., 2014), Vietnam (Nguyen et al., 2015), and Chile (Hernandez et al., 2013). ST10 was suggested as being associated with the spread of CTX-M ESBL types and *mcr-1* genes in humans, animals and environments (Monte et al., 2017). Another distinct finding is the detection of ST101 in dogs and cats in Italy. ST101 is an international sequence types frequently detected in pigs (El Garch et al., 2017), broilers (Solà-Ginés et al., 2015b) as well as in the clinical settings. In several countries, ST101 was associated to NDM-1 *E. coli* strains isolated from the clinical settings of Germany, Canada, Australia, UK, and Pakistan (Yoo et al., 2013) implying thus that ST101 is a candidate for the zoonotic transmission to the human population.

**Table 2:** ST/phylogroups, IS and plasmid types associated with beta-lactamase and *mcr* genes in the Mediterranean.

**Country**	**Animal Host**	**Species**	***Bla* and/or *mcr* genes**	**ST and/or phylogroup**	**Plasmid type**	**Associated IS**	**Reference**
Algeria	Poultry	*E. coli*	CTX-M 1	ST38, ST2179			Belmahdi et al., 2016
			SHV-12	ST1011, ST5086			
			CMY-2	ST744			
	Poultry	*S. Heidelberg*	CTX-M-1	ST15			Djeffal et al., 2017
	Cattle	*A. baumanii*	NDM-1	ST85			Chaalal et al., 2016
	Cattle	*E. coli*	NDM-5/ CMY-42/ CTX-M-15	ST1284	IncX3 (NDM-5)		Yaici et al., 2016
	Swine	*K. pneumoniae*	CTX-M-15	ST584			Bachiri et al., 2017
		*E. coli*	CTX-M 15	ST617, ST131, ST648, ST405, ST1431, ST1421, ST69, ST226			
	Dog	*E. coli*	CTX-M-15	A, B1, E			Yousfi et al., 2016b
			CTX-M-1/SHV-12	E			
			SHV-12	A, B1			
	Dog	*E. coli*	NDM-5	ST1284			Yousfi et al., 2015
	Dog	*E. coli*	OXA-48	A, D			Yousfi et al., 2016a
			NDM-5/ CTX-M-15/ CMY-42	A			
	Cat	*E. coli*	CTX-M-1	B1			Yousfi et al., 2016b
			CTX-M-15	A, U, E			
	Cat		OXA-48 / CMY-1	U			Yousfi et al., 2016a
			OXA-48	D			
	Barbary Macaques	*K. pneumoniae*	CTX-M-15	ST584			Bachiri et al., 2017
	Fish	*A. baumanii*	OXA-23	ST2			Brahmi et al., 2016
	Fish	*E. coli*	CTX-M-15	ST471, ST132, ST398, ST37,ST477, ST131, ST31			Brahmi et al., 2015
			CTX-M-9	ST8			
			TEM-24	ST31, ST471, ST66, ST21, ST74			
Tunisia	Poultry	*E. coli*	CTX-M-1	A, B1, D		IS*Ecp1*	Ben Sallem et al., 2012
			CMY-2	B2		IS*Ecp1*	
				D		IS*Ecp1D*-IS*10*	
	Poultry		CTX-M-1			IS*Ecp1*/IS*26*	Jouini et al., 2007
	Poultry	*E. coli*	CTX-M-1	B1, A			Ben Slama et al., 2010
			CMY-2	B1			
	Poultry	*E. coli*	CTX-M-1	A, B1, D, B2	IncI1		Mnif et al., 2012
			CTX-M-15	A, B1			
			CTX-M-1/CMY-2	B2	IncI1		
			CMY-2	A, D, B1	IncI1		
	Poultry	*E. coli*	CTX-M-1		IncI1		Grami et al., 2013
			CTX-M-9		IncI1		
	Poultry	*E. coli*	CTX-M-1	A0, A1, D2, B2			Kilani et al., 2015
	Poultry	*E. coli*	CMY-2	A, B1, D	IncI1, IncF, IncFIB, IncFIA		Maamar et al., 2016
			CTX-M-14	B1	IncF	IS*Ecp1*-IS*903*	
			CTX-M-1	B1, D, A	IncI1, IncF, IncFIB, IncK, IncY, IncP, IncN		
			CTX-M-15	D		IS*Ecp1*and IS*Ecp1*-IS*5*	
	Poultry	*E. coli*	CTX-M-1/*mcr-1*	D, H, K	IncHI2/ST4		Grami et al., 2016
	Poultry	*E. coli*	CMY-2/*mcr-1*	A (ST2197)	IncP (*mcr-1*)	IS*Apl1*	Maamar et al., 2018
					IncI1 (CMY-2)		
	Cattle	*E. coli*	CTX-M-1	A, B1			Ben Slama et al., 2010
			CTX-M-1/ TEM-20	B1			
	Cattle	*E. coli*	CTX-M-1			IS*Ecp1*/IS*26*	Jouini et al., 2007
			CTX-M-14			IS*Ecp1* and IS*903*	
	Cattle	*E. coli*	CTX-M-15	ST10		IS*Ecp1*	Grami et al., 2014
	Dog	*E. coli*	CTX-M-1		IncI1		Grami et al., 2013
			CTX-M-15		IncFII		
	Dog	*E. coli*	CMY-2	B1		IS*Ecp1*	Sallem et al., 2013
			CTX-M-1	D, B1, A		IS*Ecp1*	
	Cat	*E. coli*	CTX-M-1	B1, A, D		IS*Ecp1*	Sallem et al., 2013
			CTX-M-1/ TEM-135	A		IS*Ecp1* (CTX-M-1)	
	Cat	*E. coli*	CTX-M-1		IncI1		Grami et al., 2013
	Dromedaries	*E. coli*	CTX-M-1	B1		IS*Ecp1*	Ben Sallem et al., 2012
Egypt	Poultry	*E. coli*	CTX-M-15	clonal group O25b-ST131		IS*Ecp1*	Ahmed and Shimamoto, 2013
	Poultry	*E. coli*	CTX-M	A, B1, B2, D			Abdallah et al., 2015
	Poultry	*E. coli*	CTX-M-14	D			El-Shazly et al., 2017
			SHV-12	D			
			CMY-2	A, B1, D			
	Poultry	*E. coli*	*mcr-1*	phylotype A, F, B1	IncFIB; IncI1; IncI2		Lima Barbieri et al., 2017
	Cattle	*E. coli*	*mcr-1*	ST10			Khalifa et al., 2016
Lebanon	Poultry	*E. coli*	CTX-M	ST156, ST5470, ST354, ST155, ST3224			Dandachi et al., 2018a
	Poultry	*E. coli*	*mcr-1*	ST515			Dandachi et al., 2018b
	Cattle	*E. coli*	CTX-M-15	A (ST1294, ST2325, ST1303, ST4623, ST5204)			Diab et al., 2016
				B1 (ST58, ST162, ST4252, ST155, ST196, ST540)			
				D (ST69)			
			CTX-M-14	D (ST457)			
			CTX-M-15/SHV-12	A (ST10, ST2450, ST5442)			
			CTX-M-14/SHV-12	D (ST457)			
			SHV-12	A (ST218, ST617, ST5204, ST1303,ST5728,ST1140, ST746)			
	Cattle	*A. baumanii*	OXA-23	ST2			Al Bayssari et al., 2015a
		*P. aeroginosa*	VIM-2	ST1762, ST1759			
	Swine	*A. baumanii*	OXA-23	ST491			Al Bayssari et al., 2015a
	Fowl	*A. baumanii*	OXA-23	ST492, ST493			Al Bayssari et al., 2015b
			OXA-58/OXA-23	ST20			
		*P. aeroginosa*	VIM-2	ST1760, ST1761			
	Fowl	*E. coli*	OXA-48	ST38			Al Bayssari et al., 2015b
	Horse	*A. baumanii*	OXA-143	ST294			Rafei et al., 2015
	Rabbit	*A. pitii*	OXA-24	ST221			Rafei et al., 2015
Palestine	Poultry	*E. coli*	CTX-M	A, B, D			Qabajah et al., 2014
Turkey	Poultry	*E. coli*	CMY-2	A0, B2 D1, D2			Pehlivanlar Onen et al., 2015
			CTX-M-1/CMY-2	A0			
			CTX-M-1	A1, A0, D1, D2			
			CTX-M-1/SHV-5	D1			
			CTX-M-3	A0, D1			
			CTX-M-15	B1, D1, D2			
			SHV-12	D1			
			CTX-M-15/SHV-12	D2			
Italy	Poultry	*E. coli*	SHV-12		IncI1, IncFIB		Bortolaia et al., 2010
			CTX-M-1		IncI1, IncFIB, IncN		
			CTX-M-32		IncN		
	Poultry	*E. coli*	CTX-M-1		IncI1		Accogli et al., 2013
			CMY-2		IncI1		
	Poultry	*E. coli*	CTX-M	A, B1, B2, D			Ghodousi et al., 2015
			CIT like	B1, B2, D			
	Poultry	*E. coli*	CTX-M	B2, ST131			Ghodousi et al., 2016
	Swine	*E. coli*	OXA-181	B1 (ST359), A (ST641)	IncX3		Pulss et al., 2017
			*mcr-1*	A (ST641)	IncX4		
			CMY-2	A (ST641)	IncI1		
	Cat	*E. coli*	CMY	A		IS*Ecp1*/IS*26*	Bogaerts et al., 2015
	Dog	*K. oxytoca*	SHV-12, DHA-1	N.I	IncL/M		Donati et al., 2014
		*K. pneumoniae*	CTX-M-15,DHA-1	ST340	IncR (CTX-M-15)		
			CTX-M-15	ST101			
			SHV-28,	ST15			
			CTX-M-15,SHV-28,	ST15			
			CTX-M-1,SHV-28	ST15	CTX-M-1 in IncN and IncR		
			CTX-M-1	ST11			
	Cat	*K. oxytoca*	CTX-M-9	N.I	IncHI2		Donati et al., 2014
		*K. pneumoniae*	CTX-M-15, DHA-1	ST340	CTX-M-15/DHA-1 on IncR		
			SHV-28, CMY-2	ST15	CMY-2 on InCI1		
			CTX-M-15	ST101			
	Cat	*E. coli*	CTX-M-14/CMY-2	A (ST3848, ST3847)			Nebbia et al., 2014
			CTX-M-14	B2 (ST555, ST4181), B1 (ST602)			
			CTX-M-1	B2 (ST155)			
			CTX-M-15	B2 (ST131)			
Slovenia	Poultry	*E. coli*	CTX-M-1	D			Zogg et al., 2016
			SHV-12	B1 and D			
Spain	Poultry	*E. coli*	CTX-M-14	ST101, ST156,ST165, ST350, ST889, ST1137	IncK		Solà-Ginés et al., 2015b
			SHV-12	ST350, ST533	IncI1		
			CMY-2	ST429, ST131	IncK		
	Poultry	*E. coli*	CMY-2	A, D			Cortés et al., 2010
			CTX-M-14	A, B1, B2			
			CTX-M-32	A			
			CTX-M-9	B1			
			SHV-12				
			TEM-52	B1			
	Poultry	*E. coli*	CTX-M-9	O25b:H4-B2-ST131.			Mora et al., 2010
	Poultry	*E. coli*	CTX-M, SHV	A, B1, D1			Egea et al., 2012
	Poultry, Swine, Cattle	*E. coli*	CTX-M, SHV	B2, D			Doi et al., 2010
	cattle	*E. coli*	*mcr-1* /mcr-3/ CTX-M-55	ST533	non mobilizable IncHI2		Hernández et al., 2017
	Swine	*E. coli*	CTX-M-1	A			Cortés et al., 2010
			SHV-5	A			
			SHV-12	B1			
	Dog	*E. coli* (1)	CMY (1)	ST2171	IncK	IS*Ecp1*	Bogaerts et al., 2015
		*P. mirabilis* (2)	CMY (2)				
	Dog	*K. pneumoniae*	VIM-1	ST2090			González-Torralba et al., 2016
	Deer	*E. coli*	CTX-M-1	ST224	IncN	I*S26*	Alonso et al., 2016
Croatia	Mussel	*Aeromonas spp*	CTX-M-15		IncFIB		Maravić et al., 2013
France	Poultry	*E. coli*	CTX-M-1			IS*Ecp1*	Meunier et al., 2006
	Cattle	*E. coli*	CTX-M-1			IS*Ecp1*	Meunier et al., 2006
			CTX-M-15			IS*Ecp1*	
	Cattle	*E. coli*	CTX-M-15	B1		IS*Ecp1*	Valat et al., 2012
	Cattle	*E. coli*	CTX-M-1	ST2497, ST2498			Hartmann et al., 2012
			TEM-71	ST178			
	Cattle	*E. coli*	CTX-M-15,	ST2212, ST2213, ST2210, ST2214,ST2215, ST88	F31:A4:B1/IncFII F2:A–:B–/IncFII and IncI1		Madec et al., 2012
	Cattle	*K. pneumoniae*	CTX-M-14	ST45	F2:A-:B-IncFII		Dahmen et al., 2013b
		*E. coli*	CTX-M-14	ST23, ST58, ST10, ST45	F2:A-:B-IncFII		
			CTX-M-1	ST23, ST58	IncI1/ST3		
	Sheep	*K. pneumoniae*	CTX-M-15, DHA	all ST274			Poirel et al., 2013
	Swine	*E. coli*	CTX-M-1			IS*Ecp1*	Meunier et al., 2006
	Dogs	*E. coli*	CTX-M-15	A (ST410, ST617)	IncFII		Dahmen et al., 2013a
			CTX-M-1	A (ST10), B1 (ST1303, ST1249)	IncFII		
					IncFII		
	Dog	*A. baumanii*	OXA-23	ST25			Hérivaux et al., 2016
	Dogs	*E. coli*	CTX-M-1	ST345, ST1001, ST124	IncI1		Poirel et al., 2013
			CTX-M-15	NEW ST	N.T		
			TEM-52	ST359			
		*K. pneumoniae*	CTX-M-15, DHA-1	ST274			
			CTX-M-15,	ST15			
	Dogs	*E. coli*	CTX-M-1	A, B1,D	*bla*CTX-M-1/IncI1/ST3		Haenni et al., 2014b
			CTX-M-grp9	B2			
			CMY-2	A, B1, B2, D	CMY-2/IncI1/ST2		
	Dog	*E. cloacae*	CTX-M-15	ST114,ST136,ST270,ST100	IncHI2		Haenni et al., 2016c
			CTX-M-14	ST102	N.T		
			CTX-M-3	ST408	N.T		
			SHV-12	ST268	IncHI2		
	Dog	*E. coli*	CMY	ST55	N.T		Melo et al., 2017
			CMY	ST963	N.T		
			OXA-48	ST372	IncL		
	Cat	*K. pneumoniae*	CTX-M-15, DHA	ST274			Poirel et al., 2013
		*E. coli*	CTX-M-1	ST124, ST641			
			CTX-M-14	ST141			
	Cats	*E. coli*	CTX-M-15	A (ST617, ST410)			Dahmen et al., 2013a
			CTX-M-32	B1 (ST224)			
			CTX-M-3	B2 (ST493)			
			CTX-M-14	B1, (ST359), B2 (ST131)			
	Cat	*E. cloacae*	CTX-M-15	1 ST136, others ST114	IncHI2		Haenni et al., 2016c
			SHV-12	N.T	IncA/C		
	Cat	*E. coli*	CTX-M-14	ST68	IncF		Melo et al., 2017
			CTX-M-1	ST673	IncFIB		
	Cat	*A. baumanii*	OXA-23	ST1/ST231			Ewers et al., 2016
	Hedgehog	*K. pneumoniae*	CTX-M-15, DHA	ST274			Poirel et al., 2013
	Tawny Owl	*E. coli*	CTX-M-1	ST93			Poirel et al., 2013
	Domestic goose	*E. coli*	CTX-M-15	ST10			Poirel et al., 2013
	Rock pigeon	*E. coli*	CTX-M-1	ST124			Poirel et al., 2013
	Horse	*E. cloacae*	CTX-M-15	ST127, ST372, ST145, ST114	IncHI2		Haenni et al., 2016c
			SHV-12	ST135,ST145,ST118	IncHI2		
			CTX-M-1	ST268	N.T		
				ST107	IncP		
Greece	Dog	*E. coli*	CMY-2	ST212	IncI1/ST65		Vingopoulou et al., 2014

*Bla, beta-lactamase; ST, sequence type; IS, insertion sequence; N.T, non typeable*.

More deeply speaking, ESBL and AmpC encoding genes were mostly carried on conjugative IncI1, IncFIB, IncN, and IncK plasmids (Table 1). IS*Ecp1* was the most common insertion sequence associated with the CTX-M ESBL types with the main ones being *bla*CTX-M-1 and *bla*CTX-M-15 genes. IS*Ecp1* has been previously described as a potent contributor to the mobilization and insertion of *bla*CTX-M genes worldwide (El Salabi et al., 2013). As for the carbapenemase encoding genes, these latter were found to be carried by IncX3 and IncL plasmids detected in *E. coli* strains isolated from cattle, swine and dogs in Algeria, Italy, and France, respectively. Overall, the detection of a variety of sequence types and phylogroups in ESBL and AmpC producers isolated from animals of all origins within and among countries's animals suggests that the dissemination of multi-drug resistance in the Mediterranean is multi-clonal and related rather to the diffusion of conjugative plasmids carrying beta-lactamase genes.

### Prevalence of colistin resistance in livestock and domestic animals

Polymyxin E (colistin) and polymyxin B are polycationic antimicrobial peptides that are considered as the last-line antibiotic treatment for multi-drug resistant (MDR) Gram-negative bacterial infections (Olaitan and Li, 2016). From the 1960s until the 1990s, colistin was considered as an effective treatment for MDR-GNB (Olaitan et al., 2014b). However, due its nephrotoxicity within the human body, the clinical use of this antimicrobial was abandoned (Olaitan and Li, 2016). Recently, the emergence of carbapenem resistance in clinically important bacteria such as *P. aeruginosa, A. baumannii, K. pneumonia*, and *Escherichia coli*, necessitated the re-introduction of colistin into clinical practice as a last-resort treatment option (Olaitan and Li, 2016).

Colistin is not only administered in humans, its use has been also described in veterinary medicine. Indeed, it has been suggested that the uncontrolled use of colistin in animals has played an important role in the global emergence of colistin-resistant bacteria (Collignon et al., 2016). The World Health Organization recently added polymyxins to the list of critically important antibiotics used in food producing animals worldwide (Collignon et al., 2016). The main use for colistin in animals includes the treatment of gastrointestinal infections caused by *E. coli* in rabbits, pigs, broilers, veal, beef, cattle, sheep, and goats; and, in particular, gastrointestinal infections caused by *E. coli* (Poirel et al., 2017). Colistin is mainly administered orally using different formulations such as premix, powder and oral solutions (Catry et al., 2015). In European countries, several epidemiological studies reported the use of colistin in veterinary medicine. In fact, Kempf et al. reported that colistin is mainly used to inhibit infections caused by *E. coli*, a Gram-negative bacillus known as a common causative agent of diarrhea, septicemia, and colibacillosis in animals (Kempf et al., 2013). In Spain, Casal et al. revealed that colistin is among the most frequent administered drug for the treatment of digestive diseases in pigs (Casal et al., 2007).

Epidemiologically speaking, the worldwide prevalence of resistance to polymyxins accounts for 10% of Gram-negative bacteria with the highest rates being observed in Mediterranean countries and Southeast Asia (Al-Tawfiq et al., 2017). For many years, colistin resistance was thought to be mainly mediated by chromosomic mutations, with no possibility of horizontal gene transfer. However, the emergence of the *mcr-1* plasmid mediated colistin resistance gene (Liu et al., 2016) has thoroughly altered the view of colistin resistance as a worldwide problem (Baron et al., 2016). The current epidemiology of colistin resistance is poorly understood.

In the Mediterranean area (Figure 2), the first detection of *mcr-1* was in an *E. coli* strain isolated from chickens in Algeria (Olaitan et al., 2016). This same isolate was further detected in sheep in another region of this country in 2016 (Chabou et al., 2017). In Tunisia, Grami et al. reported a high prevalence of multi-clonal *E. coli* carrying the *mcr-1* gene in three chicken farms imported from France (Grami et al., 2016). Isolated strains were found to co-harbor the *bla*CTX-M-1 ESBL gene along with *mcr-1* on an IncHI2/ST4 plasmid (Table 1; Grami et al., 2016). Apart from colistin resistance, these strains were also co-resistant to tetracyclines, quinolones, fluoroquinolones, trimethoprim, and sulfonamides (Grami et al., 2016). The co-existence of ESBL and *mcr-1* genes on the same plasmid facilitates the dissemination of colistin resistant strains by the co-selective pressure applied via the use of colistin as well as possibly the utilization of non-beta-lactam antibiotics. Molecular analysis targeting the co-localization of ESBL and *mcr* genes along with the ones mediating resistance toward non-beta-lactams is however warranted in order to validate this hypothesis. Also in Tunisia, two colistin resistant *E. coli* strains positive for *mcr-1* and harboring the CMY-2 gene were recently detected in chicken. Both strains shared the same sequence type “ST2197” in addition to their PFGE patterns. The *mcr-1* gene in these latter was associated with the IS*Apl1* and was carried by IncP plasmid while the CMY-2 gene was located on an IncI1 plasmid type (Maamar et al., 2018). Furthermore, in this same country, a recent study revealed the absence of *mcr-1* and *mcr-2* positive Gram-negative bacilli in camel calves in southern Tunisia (Rhouma et al., 2018). Likewise, in Egypt, *mcr-1* was detected in *E. coli* isolated from diseased chickens as well as from cows displaying subclinical mastitis (Khalifa et al., 2016; Lima Barbieri et al., 2017). The emergence of *mcr-1* in Egypt can be related to the use of colistin in animal agriculture, and its ready application as a therapeutic agent for colibacillosis as well as other infections, in rabbits and calves (Lima Barbieri et al., 2017). In Southeast Asia, Dandachi et al. reported the detection of the *mcr-1* plasmid mediated colistin resistance gene in *E. coli* in poultry in the south of Lebanon (Dandachi et al., 2018a). This strain had a sequence type of ST515 that was not reported before in *mcr-1 E. coli* strains of poultry origin (Dandachi et al., 2018a).

Of the European countries bordering the Mediterranean, Spain was the first to report the detection of *mcr-1* in *E. coli* and *Salmonella enterica* isolated from farm animals (Quesada et al., 2016). This could be related to the fact that Spain is one of the countries were colistin is extensively used in veterinary medicine (de Jong et al., 2013). More recently, *mcr-1* co-existing with *mcr-3* on the same non mobilizable IncHI2 plasmid was detected in an *E. coli* strain recovered from cattle feces in a slaughterhouse (Hernández et al., 2017). In France, as part of routine surveillance by the French agricultural food sector, *mcr-1* was identified in four *Salmonella* spp isolated from sausage, food of poultry origin, and boot swabs taken from broiler farms (Perrin-Guyomard et al., 2016; Webb et al., 2016). *E. coli* harboring *mcr-1* was also isolated in France from pig, broiler and turkey samples (Haenni et al., 2016a). Haenni et al. reported the identification of unique IncHI2/ST4 plasmid co-localizing *mcr-1* and ESBL genes in an *E. coli* strain isolated from French veal calves (Haenni et al., 2016b). In Italy, Carnevali et al. reported the detection of *mcr-1* in *Salmonella* spp strains isolated from poultry and pigs (Carnevali et al., 2016). Subsequently, *mcr-1* was further detected in *E. coli* of swine origin. In the aforementioned report, *mcr-1* was co-existent with the carbapenemase OXA-181 in the same bacterium and was carried on an IncX4 plasmid type (Pulss et al., 2017). In the Mediterranean basin, likewise ESBL producers, *mcr* positive strains belong to different phylogroups and appear to be not clonally related; however, they were not associated to a common plasmid or an insertion sequence type. This questions the molecular mechanism by which the *mcr* genes are being disseminating in this region of the world. More molecular work is warranted in this area especially that *mcr* genes are often located on plasmids carrying ESBL and/or carbapenemase genes.

### Antibiotic use in animals and potential impact on public health

For many years, the use of antibiotics in the veterinary medicine has increased animal health via lowering mortality and the incidence of infectious diseases (Hao et al., 2014). However, in view of the heavy dissemination of resistant organisms namely ESBL, AmpC, and carbapenemase producers in addition to the emergence of colistin resistance in livestock and animals with frequent contacts with human; the efficiency of antibiotic administration to animals has been reconsidered. Indeed, antibiotic use in animals is not controlled, in that these latter are not only prescribed for treatment, but are also given for prophylaxis and as growth promoters (Economou and Gousia, 2015). In its recent publication, the world health organization recommended a reduction but an overall restriction of the use of medically important antibiotics for prophylaxis and growth promotion in farm animals (WHO, 2017). According to the world health organization list of Critically Important Antimicrobials for Human Medicine (WHO CIA list), these include mainly extended spectrum cephalosporins, macrolide, ketolides, glycopeptides and polymixins (WHO CIA, 2017). The control of antibiotic use in the veterinary sector aims to reduce the emergence of resistance in addition to preserving the efficacy of important classes for treatment in the human medicine.

In the Mediterranean region, tetracyclines, aminoglycosides, sulfonamides, fluoroquinolones, and polymixins are the most common antimicrobial classes prescribed in the veterinary sector (Table 1). The usage level of each antibiotic class in addition to its real purpose of administration apart from treatment is limited and not well understood in this area of the world. In fact, it is nowadays accepted that the over-use of antibiotics in animals is the main driven for the dissemination of multi-drug resistance (Barton, 2014). As shown in Table 1, ESBL, AmpC, and carbapenemase producers are often co-resistant to non-beta-lactam antibiotics with the most common being gentamicin, streptomycin, tetracycline, trimethoprim-sulfamethoxazole, nalidixic acid, and ciprofloxacin. One study conducted in healthy chicken in Tunisia showed the presence of *tetA, tetB, sul1*, and *sul2* on the same plasmids carrying the *bla*CTX-M genes (Maamar et al., 2016). Another study in Egypt, reported the detection of *tetB, qnrB2, qnrA1, aadA1* on the same gene cassette along with the *bla*CMY-2 AmpC beta-lactamase gene (Ahmed and Shimamoto, 2013). In Italy, *strA/B, tetD, qnrB, aadA1, sulI* genes were associated with the *bla*CTX-M and *bla*SHV ESBL genes types in companion animals (Donati et al., 2014). Furthermore, in this same country, aminoglycoside modifying enzymes (*aadA1, aadA2*), quinolone resistance genes (*qnrS1*), florfenicol/chloramphenicol resistance gene (*floR*), in addition to tetracycline and sulfonamide resistance genes (*tetA, sul1, sul2, sul3*) were found associated with OXA-48/181 and OXA-48/181/ CMY-2 /*mcr-1* positive *E. coli* strains isolated from pigs (Pulss et al., 2017). In *Salmonella enterica*, Franco et al. reported the detection of a megaplasmid harboring the *bla*CTX-M-1 ESBL gene along with *tetA, sulI, dfrA1*, and *dfrA14* conferring thus additional resistance toward tetracycline, sulfonamide, and trimethoprim (Franco et al., 2015). Beta-lactamase producing Gram-negative bacilli appear thus to be selected by the co-selective pressure applied by the use of non-beta-lactam antibiotics in livestock and companion animals. Surveillance studies addressing the types, purpose and level of antibiotic classes' administration in animals of the Mediterranean region are warranted in order to develop approaches that control the use of antibiotics while preserving animal's health. This is especially in Syria, Cyprus, Albania, Montenegro, Bosnia, Herzogovina, Monacco, Morocco, and Libya where even no data exists on the prevalence and epidemiology of multi-drug resistant organisms in animals.

The spread of multi-drug resistant organisms of animal origin is sparked by the concern of being transmitted to humans; these latter can then be causative agents for infections with limited therapeutic options (Bettiol and Harbarth, 2015). The transfer of resistant organisms from animals to humans can occur either via direct contact or indirectly via the consumption of under/uncooked animals products (Dahms et al., 2014). Recent studies have also highlighted the importance of the farms surrounding environment in the transmission chain. Air (von Salviati et al., 2015), dust (Blaak et al., 2015), contaminated waste waters (Guenther et al., 2011), and soil fertilized with animal manures (Laube et al., 2014) are all potential sources from which resistant organisms can be transferred to the general population. In their study, Olaitan et al. demonstrated the transfer of a colistin resistant *E. coli* strain from a pigs to its owner (Olaitan et al., 2015). This was documented by both strains (in the pig and its owner) having the same sequence types and sharing the same virulence as well as same PFGE patterns (Olaitan et al., 2015). The increased risk of ESBL fecal carriage in humans with frequent contact with broilers has been further taken as an evidence of transmission (Huijbers et al., 2014). Furthermore, sharing the same sequence types, virulence and PFGE patterns in addition to common plasmids/ESBL genes are all proofs for the possible transfer of resistant organisms and/or genes from the veterinary sector to the human population (Leverstein-van Hall et al., 2011). In Algeria, Djeffal et al. reported the detection of a common sequence type (ST15) in *Salmonella spp* producing ESBL isolated from both humans and avian isolates (Djeffal et al., 2017). In Egypt, Hamza et al. showed an abundance of carbapenemase genes namely *bla*OXA-48, *bla*KPC and *bla*NDM in chicken, drinking water, and farm workers suggesting a possible transmission of carbapenemase encoding genes from broilers to farmers and the surrounding environment (Hamza et al., 2016). Another study conducted in Italy reported the spread of a multi-drug resistant clone of “*Salmonella enterica* subsp. enterica serovar Infantis” that was first detected in 2011 in broiler farms and few years later led to human infections most likely via transmission from the broiler industry (Franco et al., 2015). In Spain, common *bla*CTX-M-grp1 and *bla*CTX-M-grp9 ESBL genes were detected in retail meat as well as in *E. coli* strains isolated from infected and colonized patients in the same region (Doi et al., 2010). In France, Hartmann et al. showed a clonal relationship among CTX-M carrying *E. coli* strains in cattle and farm cultivated soils (Hartmann et al., 2012). Another study in cattle, demonstrated that CTX-M-15 harboring plasmids in non-ST131 *E. coli* strains are highly similar to those detected in humans suggesting thus a multi-clonal plasmidic transmission of multi-drug resistant organisms from livestock to the humans (Madec et al., 2012). The detection of common genes and sequence types among animals and humans and the surrounding environment emphasizes the need to have a global intervention measures to avoid the dissemination of multi-drug resistance in the one health concept.

## Conclusion

Antimicrobials have been used in veterinary medicine for more than 50 years. The use of antibiotics proved to be crucial for animal health by lowering mortality and incidence of diseases, in addition to controlling the transmission of infectious agents to the human population. Recently, the dissemination of ESBL, carbapenemase, and colistin resistant Gram negative bacteria in food producing animals brought into question the real efficacy of antibiotic administration in animals in terms of treatment, prophylaxis and growth promotion. Indeed, the emergence of MDR in food producing animals has been suggested to be largely linked to the over and misusage of antibiotics in veterinary medicine. The level of antibiotic consumption in animals varies between countries. Although, cephalosporins are not often prescribed in veterinary medicine, the use of other non-beta-lactams could account for the co-selection of multi-drug resistant bacteria. As shown in Table 1, ESBL and carbapenemase producers were frequently co-resistant to aminoglycosides, tetracyclines and fluoroquinolones, with these latter being mostly used in the veterinary field. Furthermore, the aforementioned antibiotics are classified by the World Health Organization as critically important antibiotics for human medicine that should be restricted in the animal field (Collignon et al., 2016). That said, the direct public health effect of the transmission of MDR bacteria from animals to humans is still controversial. Several studies have demonstrated a direct link of transmission between these two ecosystems. Resistant bacteria once transmitted to humans can be further selected by the over-use of antimicrobial agents in the clinical and community settings. This spread will promote the global dissemination of bacterial resistance across all ecosystems. The level of antibiotic consumption in animals in the European countries lining the Mediterranean is available in the European Surveillance of Veterinary Antimicrobial Consumption report (EMA/ESVAC, 2014), however this is not the case for the countries in North Africa and western Asia, where no accurate data are available. Therefore, surveillance studies investigating the levels of antibiotic prescription should be conducted in these areas. Antimicrobial prescriptions in animals should be re-considered and controlled to limit the spread of bacteria which are cross resistant to the antibiotics used in human medicine. In addition, a risk assessment of other factors contributing to the emergence of antimicrobial resistance in animals should be conducted in future studies. Poor sanitary conditions, overcrowding and poor infection control practices in animals are all possible contributors to the robust emergence of MDR in food-producing animals.

## Author contributions

ID and SC wrote the review paper. ZD and J-MR corrected the manuscript. All authors approved and revised the final version of the manuscript.

### Conflict of interest statement

The authors declare that the research was conducted in the absence of any commercial or financial relationships that could be construed as a potential conflict of interest.
